# Supplementary material for: Bayesian refinement of protein structures and ensembles against SAXS data using molecular dynamics
Source: PLoS Comput Biol. 2017 Oct 18;13(10):e1005800. doi: 10.1371/journal.pcbi.1005800 (PMC5662244; doi:10.1371/journal.pcbi.1005800)
Supplement: S1 Archive — (BZ2) [file pcbi.1005800.s011.tar.bz2 › gromacs_4.62_bayesian_saxs_refi/share/html/online/genrestr.html]

genrestr


| genrestr Main Table of Contents |

**VERSION 4.6  
Sat 19 Jan 2013**

---

### Description

genrestr produces an include file for a topology containing
a list of atom numbers and three force constants for the
x-, y-, and z-direction. A single isotropic force constant may
be given on the command line instead of three components.

WARNING: position restraints only work for the one molecule at a time.
Position restraints are interactions within molecules, therefore
they should be included within the correct [ moleculetype ]
block in the topology. Since the atom numbers in every moleculetype
in the topology start at 1 and the numbers in the input file for
genrestr number consecutively from 1, genrestr will only
produce a useful file for the first molecule.

The -of option produces an index file that can be used for
freezing atoms. In this case, the input file must be a .pdb file.

With the -disre option, half a matrix of distance restraints
is generated instead of position restraints. With this matrix, that
one typically would apply to Cα atoms in a protein, one can
maintain the overall conformation of a protein without tieing it to
a specific position (as with position restraints).

### Files

| option | filename | type | description |
| --- | --- | --- | --- |
| **-f** | conf.gro | Input | Structure file: gro g96 pdb tpr etc. |
| **-n** | index.ndx | Input, Opt. | Index file |
| **-o** | posre.itp | Output | Include file for topology |
| **-of** | freeze.ndx | Output, Opt. | Index file |

### Other options

| option | type | default | description |
| --- | --- | --- | --- |
| **-[no]h** | bool | no | Print help info and quit || **-[no]version** | bool | no | Print version info and quit || **-nice** | int | 0 | Set the nicelevel || **-fc** | vector | 1000 1000 1000 | Force constants (kJ/mol nm^2) || **-freeze** | real | 0 | If the -of option or this one is given an index file will be written containing atom numbers of all atoms that have a B-factor less than the level given here || **-[no]disre** | bool | no | Generate a distance restraint matrix for all the atoms in index || **-disre\_dist** | real | 0.1 | Distance range around the actual distance for generating distance restraints || **-disre\_frac** | real | 0 | Fraction of distance to be used as interval rather than a fixed distance. If the fraction of the distance that you specify here is less than the distance given in the previous option, that one is used instead. || **-disre\_up2** | real | 1 | Distance between upper bound for distance restraints, and the distance at which the force becomes constant (see manual) || **-cutoff** | real | -1 | Only generate distance restraints for atoms pairs within cutoff (nm) || **-[no]constr** | bool | no | Generate a constraint matrix rather than distance restraints. Constraints of type 2 will be generated that do generate exclusions. |

---

http://www.gromacs.org  
gromacs@gromacs.org
